# Retracted: The Healing of Oxidative Injuries with Trehalose in UVB-Irradiated Rabbit Corneas

**DOI:** 10.1155/2020/4128985

**Published:** 2020-12-19

**Authors:** Oxidative Medicine and Cellular Longevity

*Oxidative Medicine and Cellular Longevity* has retracted the article titled “The Healing of Oxidative Injuries with Trehalose in UVB-Irradiated Rabbit Corneas” [1] due to concerns with the figures. We were contacted by an author of the article who identified that Figure 4c in [1] is duplicated with 6a in [2], despite different experimental conditions being described. Figure 6c in [1] is also duplicated with Figure 5d in [2] despite different experimental conditions being described. The first author responded with an explanation and revised figures, but these did not satisfactorily address the concerns of the editorial board. The article is therefore being retracted due to concerns regarding the reliability of the data. The authors Sarka Kubinova and Céline Olmiere agree to the retraction of the article, however, all other authors do not agree.
